# Magnetic Resonance Imaging in a Patient with a Dual Chamber Pacemaker

**DOI:** 10.1155/2010/292071

**Published:** 2011-02-06

**Authors:** Lynne Martina Millar, Andrew George Robinson, Maurice Thomas O'Flaherty, Niall Eames, Nicola Johnston, Gary Heyburn

**Affiliations:** ^1^Department of Cardiology, Royal Victoria Hospital, 274 Grosvenor Road, Belfast BT12 6BA, Northern Ireland; ^2^Department of Trauma and Orthopaedics, Royal Victoria Hospital, 274 Grosvenor Road, Belfast BT12 6BA, Northern Ireland

## Abstract

Having a pacemaker has been seen an absolute contraindication to having an MRI scan. This has become increasingly difficult in clinical practice as insertion of pacemakers and implantable cardiac defibrillators is at an all time high. Here we outline a case where a 71-year-old male patient with a permanent pacemaker needed to have an MRI scan to ascertain the aetiology of his condition and help guide further management. Given this clinical dilemma, an emergency clinical ethics consultation was arranged. As a result the patient underwent an MRI scan safely under controlled conditions with a consultant cardiologist and radiologist present. The results of the MRI scan were then able to tailor further treatment. This case highlights that in certain conditions an MRI can be performed in patients with permanent pacemakers and outlines the role of clinical ethics committees in complex medical decision making.

## 1. Introduction

Magnetic resonance imaging (MRI) is the gold standard imaging modality for the investigation of suspected intracranial or musculoskeletal pathology with 30 million MRI scans being performed per annum worldwide [1]. It allows excellent soft tissue delineation with minimal contrast toxicity and no radiation exposure. In parallel to the increasing use of MRI is the increase in the use of cardiac devices such as pacemakers and implantable cardiac defibrillators. Each year 80,000 patients in the United States undergo pacemaker insertion [2]. In the past having a pacemaker in situ was seen as an absolute contraindication to having an MRI scan. In more recent years there have been a limited number of trials which have shown that in certain cases a patient with a pacemaker can undergo an MRI scan.

Here we report a case of a patient who underwent an MRI scan with a pacemaker in situ and explore the use of clinical ethics committees in medical decision making.

## 2. Case Report

A 71-year-old retired architect presented with an 18-month history of intermittent headaches, difficulty in swallowing, and neck pain. He did not suffer any recent weight loss. Although he was not pacemaker dependent, he previously had a permanent pacemaker inserted for symptomatic sinus bradycardia four years previously. Other past medical history included transurethral resection of the prostate. He took no regular medications but had been using nonsteroidal anti-inflammatory drugs as analgesia for his neck pain. Examination of the central and peripheral neurological systems was normal. He had a slight decrease in global neck movements due to pain at the extremes of motion. Plain x-rays of the cervical spine showed some mild degenerative changes and generalised osteopenia but few other abnormalities.

Computed tomography (CT) scanning of the brain and cervical spine identified extensive destruction of the base of the skull centred on the clivus with abnormality of the occipitocervical junction. It was felt that these appearances were possibly due to metastases or multiple myeloma. A CT scan of the chest, abdomen, and pelvis was performed but no significant abnormality was detected. A bone scan confirmed a solitary lesion at the base of the skull. Bence Jones proteins were negative and globulins were within the normal range. Thyroid function and prostate specific antigen were normal. A full-body positron emission tomography (PET) scan did not identify any significant abnormalities.

A clinical dilemma therefore existed. If this was a primary tumour of the brain with bony destruction, the prognosis may be very guarded and major surgery might not be appropriate. Alternatively if the tumour originated from the pituitary gland, surgical treatment may be possible and potentially curable. 

Given the instability of the occipitocervical junction, occipitocervical fusion of C2/C3/C4 to the occiput was felt to be necessary in the first instance. After informed consent was obtained this was performed without complication.

After this, it was felt that an MRI scan was essential to characterise the lesion and to plan further intervention. The radiology department staff were clearly apprehensive to proceed with an investigation which they felt could potentially have fatal consequences. They had never conducted an MRI scan in a patient with a pacemaker and therefore were keen to seek advice on the ethics of carrying out such a procedure. Therefore an emergency clinical ethics consult was arranged. The emergency ethics consult covered a number of key areas including the effect of turning off the pacemaker on the patient and the effect of an MRI scan on the pacemaker with the possible need for urgent pacemaker replacement. The possibility of damage to the MRI scanner was also considered as well as associated health and safety issues to radiology staff. Alternative imaging modalities were discussed as was the evidence base available for MRI scanning of patients with pacemakers in situ. It was felt there was no suitable alternative imaging modality to MRI. The patient was counselled regarding the risks involved and the reasons as to why it was felt necessary. The patient's medical team felt the ethical input greatly clarified their thinking. 

Therefore after informed consent, an MRI scan was organised. The MRI scanner that was used was a Phillips Achivea with a static field strength of 1.5 Tesla. The patient's pacemaker was a Medtronic pacemaker in DDD mode set at 60–150 bpm. There was a bipolar atrial lead and a unipolar ventricular lead. Under the direct supervision of a consultant cardiologist the pacemaker was turned to OAO mode. The pacing check prior to the MRI showed pacing parameters and battery voltage within the normal range. The patient's observations including blood pressure, heart rate, oxygen saturation, and ECG monitoring were normal throughout the MRI. The patient's intrinsic rhythm was sinus rhythm and it remained unchanged throughout. The MRI sequences included sagittal T1, axial T2, axial T2 FLAIR, axial, and sagittal T1 postgadolinium. The pacing parameters were checked after the MRI, and they were unchanged from the pre-MRI parameters. There was no increased impedence and the thresholds were the same. The pacemaker was then reset to pre-MRI settings. After MRI there was no raise in cardiac troponin levels. There was no apparent effect of the pacemaker on the MRI scanner, and the MRI images produced were of good quality. 

The MRI revealed destruction of the skull base with a significant loss of the structural integrity of the occipitocervical junction. It revealed that the tumour appeared to be arising from the medulla of the skull base and it was potentially treatable. The patient then underwent an anterior approach to the skull base transorally. Samples revealed tissue in keeping with a plasmacytoma, and haematological treatment was commenced with the patient to date recovering well.

This case highlights two important points: firstly that a pacemaker may not be an absolute contraindication to MRI scanning and secondly the important role of Clinical Ethics Committees in difficult medical decisions like this one.

## 3. Discussion

The evidence around pacemakers being a contraindication to MRI is controversial. Some of the earlier lines of evidence is gathered anecdotally from a few patients who died after an MRI scan and in vitro studies performed using older pacemaker technology and leads. Within the last decade there have been a small number of trials that have demonstrated pacemaker patients undergoing MRI scanning safely. The risks associated with performing an MRI in a patient with a pacemaker include motion, dislocation, changes to programming, changes in the pacemaker components caused by static/pulsed magnetic field, and interference of time-varying gradient magnetic field with pacemaker function which mimics intrinsic cardiac activity and heating [1, 3]. 

We know of at least 10 known cases of deaths related to MRI scanning in patients with pacemaker in situ during the late 1980s [4]. Inrich et al. searched for all the cases reported in Germany during 1992–2001. They discovered six fatalities for which the German public prosecutor had ordered postmortem examinations on. These MRI scans had been performed in private radiology practices for neurosurgical or orthopaedic reasons but there did not appear to be any cardiac monitoring during the procedure. The postmortem results on the 3 of the cases concluded that the underlying cause of death was presumed ventricular fibrillation [5]. Both Roguin et al. [6] and Martin et al. [7] strongly recommend a physician competent in pacing programming should be present during the procedure. For these reasons we carefully monitored our patient's ECG recording and vital signs during the MRI and had a consultant cardiologist present for the procedure. 

Some of the risks of MRI scans in patients with pacemakers will now be explored in further detail. One of the concerns regarding MRI scans in those with pacemakers is the potential for lead heating. Sommer et al. found that the temperature increase was related to the specific absorption rate (SAR) with 8.9°C at 0.6 W/kg and an increase of 23.5°C with an SAR of 1.3 W/kg. The increase was more marked when the lead loop was placed near/at the centre of the body coil [2]. There is also the potential for a pacemaker to undergo electrical reset. Sommer et al. found that 7 of the 115 MRI scans performed were found to have gone through electrical reset although this was not found to be clinically significant [8]. Roguin et al. found that a third of their pacemakers also went through electrical reset but these were all of the same model [6]. This could lead to potential bradyarrhythmias or inadequate pacemaker function in those who are pacemaker dependent. For this reason it makes MRI scans much more dangerous in these patients. In our patient we turned the pacemaker to OAO mode thus preventing potential arrhythmias. Reprogramming the pacemaker as we did to “therapy off” in nonpacemaker dependent patients is recommended by Roguin et al. [6]; however, Martin et al. feel this is unnecessary as the pacemaker will automatically enter asynchronous mode in the static magnetic field [7]. Reduction in battery voltage had also been reported. One study investigating MRI scans at field strengths of 0.5 Tesla (T) found that there was reduction in battery voltage but this returned normal at three-month review [2] whereas the same author found in a different study at 1.5 T that this was statistically significant [8]. However it was felt that the changes were minor enough not to affect the longevity of the pacemaker dramatically [8]. One could argue that providing battery voltage was checked after an MRI scan; this alone would not preclude the examination. In our patient battery voltage remained unchanged before and after MRI scan.

## 4. Clinical Ethics Committees

This case outlines the important role that Clinical Ethics Committees (CEC) can have in difficult medical decision making. Ethical committees have been established for decades within the United States; however these are new phenomena within the UK [9]. As a result some clinical staff may not be aware of their existence. They provide education and training, policy formation, and consultation on difficult cases. They can provide an impartial body which can provide guidance and advice to physicians. In this case there was obvious clinical dilemma for which there was no clear solution. The involvement of the CEC was not to provide legal cover but to ensure the decision making followed a logical ethical framework. This was an extraordinary event for the teams involved, and all parties welcomed the involvement of the committee and the reassurance that came from their involvement.

## 5. Conclusion

This paper outlines a case where a patient with a dual chamber pacemaker successfully underwent an MRI scan at 1.5 T without complication. It demonstrates the support a clinical ethics committee can provide to physicians when they are faced with an ethical dilemma. Although this paper does not endorse the routine use of MRI scans in patients with pacemakers, it merely highlights it may be possible in controlled conditions. With pacemaker insertion being at an all-time high this may become more frequent in the future.
